# Plasmacytoid Dendritic Cells Accumulate and Secrete Interferon Alpha in Lymph Nodes of HIV-1 Patients

**DOI:** 10.1371/journal.pone.0011110

**Published:** 2010-06-14

**Authors:** Clara Lehmann, Mark Lafferty, Alfredo Garzino-Demo, Norma Jung, Pia Hartmann, Gerd Fätkenheuer, Jeffrey S. Wolf, Jan van Lunzen, Fabio Romerio

**Affiliations:** 1 Institute of Human Virology, University of Maryland, Baltimore, Maryland, United States of America; 2 Department of Medicine, University of Maryland School of Medicine, Baltimore, Maryland, United States of America; 3 First Department of Internal Medicine, University of Cologne, Cologne, Germany; 4 Department of Microbiology and Immunology, University of Maryland School of Medicine, Baltimore, Maryland, United States of America; 5 Department of Otorhinolaryngology-Head and Neck Surgery, University of Maryland Medical Center, Baltimore, Maryland, United States of America; 6 University Medical Center Hamburg-Eppendorf and Heinrich-Pette-Institute for Experimental Virology and Immunology, Hamburg, Germany; Karolinska Institutet, Sweden

## Abstract

Circulating plasmacytoid dendritic cells (pDC) decline during HIV-1 infection, but at the same time they express markedly higher levels of interferon alpha (IFNα), which is associated with HIV-1 disease progression. Here we show an accumulation of pDC in lymph nodes (LN) of treatment-naïve HIV-1 patients. This phenomenon was associated with elevated expression of the LN homing marker, CCR7, on pDC in peripheral blood of HIV-1 patients, which conferred increased migratory capacity in response to CCR7 ligands in *ex vivo* functional assays. LN-homed pDC of HIV-1 patients presented higher CD40 and lower BDCA2 levels, but unchanged CD83 and CD86 expression. In addition, these cells expressed markedly higher amounts of IFNα compared to uninfected individuals, and were undergoing faster rates of cell death. These results demonstrate for the first time that in asymptomatic, untreated HIV-1 patients circulating pDC up-regulate CCR7 expression, accumulate in lymph nodes, and express high amounts of IFNα before undergoing cell death. Since IFNα inhibits cell proliferation and modulates immune responses, chronically high levels of this cytokine in LN of HIV-1 patients may impair differentiation and immune function of bystander CD4^+^ T cells, thus playing into the mechanisms of AIDS immunopathogenesis.

## Introduction

Untreated HIV-1 infection is characterized by a progressive decline of CD4^+^ T cell number and function. Recent studies have shown that CD4^+^ T cells are rapidly depleted from the gut-associated lymphoid tissue (GALT) and other mucosal sites within the first few weeks of infection (for a comprehensive review see [1]). Mucosal depletion of CD4^+^ T cells is followed by the onset of generalized immune activation and progressive loss of immune function, which are manifested throughout the asymptomatic chronic phase of the disease [1]. The level of immune activation in HIV-1 infected subjects is a strong independent predictor for HIV-1 disease progression [2], [3], [4], [5], [6]. However, the mechanisms that trigger and drive persistent immune activation remain to be elucidated.

Interferon alpha (IFNα) is rapidly upregulated in response to viral infections [7], [8], [9], and is an essential player in antiviral immune responses: it induces the expression of cellular genes that interfere with viral replication, activates NK cell function, and promotes maturation of antigen presenting cells [10], [11], [12], [13], [14]. Plasmacytoid dendritic cells (pDC) are the main IFNα producers in humans [15].

The role of pDC and IFNα during HIV-1 infection has been a matter of intense debate. Indeed, HIV-1 disease progression is associated with a decline of pDC from the peripheral compartment, which correlates with high viral load and reduced CD4 counts [16]. In addition, pDC of HIV-1 patients produce lower levels of IFNα when challenged *ex vivo* with reference viruses [16], [17], [18], [19], [20], [21], [22]. On the other hand, several studies have documented the chronic production of IFNα in HIV-1 patients, which is associated with disease progression [23], [24], [25], [26]. Our and other groups showed that increased IFNα expression correlates with HIV-1/SIV disease progression [27], [28], [29]. In particular, we showed that a specific subtype of IFNα – namely IFNα2b – is preferentially up-regulated in HIV-1 patients throughout the course of the disease [30]. Moreover, we demonstrated that – despite their decline – residual pDC in peripheral blood of HIV-1 patients express IFNα at levels markedly higher than pDC of healthy controls [28]. Recent studies have suggested that IFNα may play a role in driving immune activation during HIV-1 infection. Siliciano and colleagues reported the up-regulation of type I interferon-regulated genes – including cell cycle-associated genes – in activated CD4+ T cells of HIV-1 patients [31]. Moreover, IFNα promotes apoptosis of uninfected, bystander CD4^+^ T cells [32], and gene expression profiles suggest that exposure to IFNα may induce chronic activation of CD4^+^ T cells [31]. Evidence for a possible role of pDC and IFNα in AIDS pathogenesis also comes from animal models. Persistent decline of circulating pDC and chronic over-expression of IFNα are observed during pathogenic SIV infection of non-natural hosts [33], [34], but not during non-pathogenic SIV infection of natural hosts [29], [35]. These reports suggest that IFNα may play a role involved in promoting chronic activation and immune dysfunction during HIV-1 disease.

Although various hypotheses have been called into play [19], [36], [37], [38], [39], [40], one key aspect that remains to be elucidated is whether the decline of pDC observed in peripheral blood of HIV-1 patients reflects systemic depletion or relocation to other body compartments. Thus, in the present study we investigated the fate and function of pDC in HIV-1 infection. We analyzed the expression of tissue homing markers on circulating pDC from HIV-1 patients and control individuals. Further, we analyzed frequency, maturation stage, steady-state IFNα secretion, and cell death rate of pDC in lymph nodes of HIV-1 patients and healthy subjects. Our results show that circulating pDC of HIV-1 patients relocate to lymphoid tissues and express high levels of IFNα before undergoing cell death.

## Materials and Methods

### Patients

All samples were obtained with signed informed consent after approval from the Institutional Review Boards of the University of Maryland, Baltimore and University of Hamburg, Germany, and according to ethical guidelines. Lymph nodes (cervical and axillary) and peripheral blood were obtained from HIV-1 patients and uninfected controls at the University of Hamburg-Eppendorf, Germany, at the University of Maryland School of Medicine, at the University of Maryland Medical Center, and at the National Disease Resource Interchange Program (Philadelphia, PA). Lymph nodes were placed in sterile saline immediately after excision, and mononuclear cells were dissociated mechanically. Peripheral blood and lymph node mononuclear cells (PBMC and LNMC) were isolated by Ficoll centrifugation. Frozen PBMC and LNMC samples were thawed and analyzed all at the same time at the Institute of Human Virology (Baltimore, MD). To ensure reliability of the functional measures carried out with cryopreserved samples – including the exclusion of samples that presented ≥25% of dead cells by trypan blue exclusion upon thawing – we followed guidelines reported in several studies published previously [41], [42], [43], [44].

### Flow Cytometry

For surface staining, PBMC and LNMC suspensions were incubated in the dark for 30 minutes at 4°C with anti-CD123 and -BDCA2 or -BDCA4 antibodies (Miltenyi Biotec, Auburn, CA) to identify pDC, in combination with anti-CCR4, -CCR7, -CCR9, -CD18, -CD29, -CD40, -CD49d, -CD83, -CD86, -CD62L, -CD103, -Integrin β7, or Annexin V (all from BD Biosciences, San Jose, CA). As control, we used isotype-matched antibodies labeled with the appropriate fluorochrome (BD Biosciences). After staining, cells were washed with phosphate buffer saline (PBS) and analyzed by flow cytometry (FACSCalibur) using CellQuest software (both from BD Biosciences). In all cases, 10^6^ events were acquired corresponding to live mononuclear cells as assessed by forward and side light scatter profile. Steady state IFNα secretion was assayed using the IFNα Secretion and Detection Kit (Miltenyi Biotech). Briefly, immediately after thawing LNMC were incubated with an anti-IFNα Catch Reagent (anti-IFNα antibody conjugated to a proprietary cell surface-specific monoclonal antibody). After a 20-minute incubation to allow IFNα secretion and capture by the Catch Reagent, the cells were incubated with an IFNα Detection Reagent (PE-labeled anti-IFNα antibody) along with antibodies to CD123 and BDCA2 to identify pDC by flow cytometry. Expression of the homing markers, staining with Annexin V and IFNα secretion were determined as Geometric Mean Fluorescence Intensity (GMFI) by gating on the live cell population (as assessed by forward and side scatter light profile) and on the pDC population (as assessed by BDCA2/4^+^CD123^+^). Data analysis was performed with the FlowJo software (Tree Star, Ashland, OR).

### Migration Assays

Evaluation of migratory properties of pDC from peripheral blood of HIV-1 positive and negative subjects was performed using a modified transmigration assay [45]. This assay utilized unfractionated PBMC, thereby avoiding unintentional *in vitro* maturation effects during purification, and increased total input PBMC concentration to account for decreased frequencies of dendritic cells in PBMC of HIV-1 patients. Briefly, CCL19 (MIP3β) and CCL21 (SLC) (R & D Systems, Minneapolis, MN) were diluted to 100 ng/ml each in RPMI 1640 supplemented with 10% human serum AB, and added (alone or in combination) in the lower chamber of a 24-well Costar transwell plate (Corning Inc., Lowell, MA). Media without chemokine was used as control. PBMC were placed at 2×10^7^ cells/ml in RPMI plus 10% human serum AB, 100 µl (2×10^6^ cells) were added to the upper chamber and incubated for 2 hours at 37°C in 5% CO_2_. Cells in the bottom wells were then harvested by pipetting, and residual cells were collected by rinsing the underside of the filter with 10 mM EDTA in PBS. Cells were washed and stained with the appropriate antibodies, and analyzed by flow cytometry. Duplicate results were averaged. Net migration was defined as the percentage of pDC migrating in response to chemokines minus the percent migrating in media alone.

### Real-time PCR Quantification of HIV-1 DNA

Total cellular DNA was extracted from 1×10^6^ LNMC using the DNeasy Blood and Tissue Kit (Qiagen, Valencia, CA). DNA was analyzed by real-time quantitative PCR using a Bio-Rad iQ5 Real-Time PCR system with iQSYBR green supermix (Bio-Rad, Hercules, CA). Reactions were performed in triplicate in parallel with sets of known quantitative standards for HIV-1 DNA and albumin. HIV-1 DNA was quantified using the following primers 5′-GGCTAACTAGGGAACCCACTG-3′ (sense) and 5′-CTGCTAGAGATTTTCCACACTGAC-3′ (antisense). PCR was performed for 2 min at 95°C followed by 40 cycles of 30 sec at 95°C and 30 sec at 60°C. Cell number was determined using albumin as a reference with the following primers 5′-TGTTGCATGAGAAAACGCCA-3′ (sense) and 5′-GTCGCCTGTTCACCAAGGAT-3′ (antisense). PCR was performed for 2 min at 95°C followed by 40 cycles of 30 sec at 95°C and 1 min at 62°C. The viral load was expressed as percent of CD4^+^ T cells harboring HIV-1 genome.

### Statistical Analyses

Data were analyzed with Wilcoxon rank-sum test. Spearman's *r* was used to describe correlations. All statistical analyses assumed a 2-sided significance level of 0.05. Data were summarized using Median and Interquartile Range (IQR) or Mean ± Standard Error of Means (SEM), as indicated in the tables and figure legends. Data analyses were performed using GraphPad Prism (GraphPad Software, San Diego, CA).

## Results

### Accumulation of pDC in lymph nodes of HIV-1 patients

Recent studies strongly suggest that chronic, exacerbated IFNα expression may be a cofactor in the immunopathogenesis of HIV-1 disease [29], [31]. As upon virus infection pDC are the main IFNα producers, a better understanding of their fate and function during HIV-1 infection may help elucidate the mechanisms of immune dysfunction. Previous studies showed a decline of pDC in peripheral blood of HIV-1 patients [18], [28]. Thus, we sought to assess whether this observation could be explained with relocation to lymph nodes. To that end, we obtained lymph node samples from HIV-1 patients (*n* = 18) and healthy controls (*n* = 11), whose demographic and clinical characteristics are shown in Table 1. The median age (and interquartile range, IQR) of HIV-1 positive individuals at time of sample collection was 31 years (26–35) and 100% were male Caucasians. The median age for uninfected individuals was 50 years (26–61), 73% were male, and 81% were Caucasians. HIV-1 patients had a median CD4^+^ T cell count of 413 cells/µl (348–538), median viremia of 4.5 log_10_ copies of HIV-1 RNA per ml of blood (3.7–5.3), while we determined that a median 1.3% of CD4^+^ T cells in lymph node mononuclear cells carried proviral HIV-1 DNA (0.2–1.5). All HIV-1 patients were off therapy at the time of sample collection. In addition, as indicated in Materials and Methods, we followed previously described guidelines – e.g. the exclusion of samples with ≥25% of dead cells by trypan blue exclusion upon thawing – so as to ensure the reliability of studies carried out with cryopreserved samples [41], [42], [43], [44].

**Table 1:** Demographic and clinical parameters of lymph node donors.

Characteristic	HIV-1 positive (*n* = 18)	Controls (*n* = 11)
Age	31 (26–35)	50 (26–61)
Gender (Male: Female)	18∶0	8∶3
Ethnicity (Cauc. : Afr. Amer. : Hisp.)	18∶0∶0	9∶1∶1
CD4^+^ T cells/µl	413 (348–538)	n.a.
Percent pDC in total LNMC	0.87±0.08	0.45±0.05
Log_10_ HIV-1 RNA copies/ml blood	4.5 (3.7–5.3)	N/A
Percent HIV-1^+^ CD4^+^ T cells in LN	1.3 (0.2–1.5)	N/A
ART status (On: Off)	0∶18	N/A

All data are Median (Interquartile Range) except for % pDC in total LNMC, which is Mean ± Standard Error of Means; n.a.  = not available; N/A = not applicable; LNMC = lymph node mononuclear cells; ART = Antiretroviral therapy.

**Figure 1:**
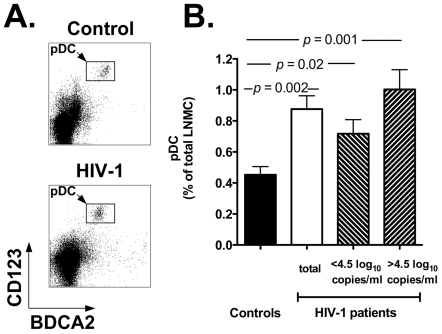
Accumulation of pDC in lymph nodes of HIV-1 patients. Panel A: flow cytometry plots showing the pDC population in lymph node mononuclear cells (LNMC) of a representative control individual (top) and HIV-1 patient (bottom). We identified pDC by staining whole LNMC with anti-BDCA2 and anti-CD123 antibodies (rectangles within density plots) and by gating onto the live cell population as assessed by forward and side light scatter profile (not shown). Panel B: summary results for the frequency of pDC in LNMC of healthy controls (black bar, *n* = 11) and HIV-1 patients (white bar: total patients, *n* = 18; downward diagonal bar: patients with <4.5 log_10_ copies/ml HIV-1 RNA, *n* = 9; upward diagonal bar: patients with >4.5 log_10_ copies/ml HIV-1 RNA, *n* = 9). The panel shows the Mean and SEM values of pDC frequencies for each study group.

We analyzed the frequency of pDC in lymph node mononuclear cells (LNMC). Figure 1A reports two flow cytometry plots representative of a healthy control (top plot) and an HIV-1 positive individual (bottom plot), while Figure 1B shows the summary results for all individuals in each study group (black bar: controls; white bar: HIV-1 patients). Our results show a significant increase in the percentage of lymph node-homed pDC in HIV-1 patients compared to healthy controls (*p* = 0.002). Indeed, the mean percentage of pDC in LNMC of HIV-1 infected patients was 0.87±0.08 vs. 0.45±0.05 in control individuals. Moreover, when HIV-1 patients were stratified around the median value of the HIV-1 viral load (4.5 log_10_ copies/ml), the difference in the percentage of LN-homed pDC between control individuals and HIV-1 patients with viremia >4.5 log_10_ copies/ml was even more remarkable (*p* = 0.001; Figure 1B; upward diagonal bar). No statistically significant difference was observed between HIV-1 patients with viremia >4.5 log_10_ and <4.5 log_10_ copies/ml (*p*>0.05). These results suggest that in HIV-1 positive individuals circulating pDC relocate from the peripheral blood to lymph nodes, possibly in response to undergoing viral replication. It is also important to underscore that for these and all the other results reported below, all samples were analyzed concurrently, side by side to ensure reproducibility of the data.

### Increased expression of the lymph node homing markers CCR7 and CD62L, and GALT homing marker CD103 on circulating pDC of HIV-1 patients

In an attempt to elucidate the mechanisms involved in the accumulation of pDC in lymph nodes of HIV-1 patients, we investigated the expression of lymph node and other tissue homing markers on circulating pDC of 17 HIV-1 positive and 11 uninfected individuals in a cross sectional study.

Table 2 reports the demographic and clinical parameters of the peripheral blood donors. The median age (and IQR) of HIV-1-infected individuals at time of sample collection was 41 years (36–51) and ∼70% were male African American, while for uninfected individuals was 37 years (31–39), 55% were male, and 64% were Caucasians. For HIV-1 positive subjects, the median absolute CD4^+^ T cell count (and IQR) was 402 cells/µl (294–530), and the median HIV-1 RNA copies/ml blood was 3.7 log_10_ (1.7–4.7). The percentage of pDC in PBMC of HIV-1 positive individuals was markedly reduced compared to negative controls: 0.25±0.04 vs. 0.57±0.07; *p* = 0.003. About two thirds of the HIV-1 patients were off antiretroviral therapy at the time of sample collection. As described previously, the decline in pDC frequency correlated inversely with HIV-1 viremia (*p* = 0.01, *r* = −0.7; data not shown) [28].

**Table 2:** Demographic and clinical parameters of peripheral blood donors.

Characteristic	HIV-1 positive (*n* = 17)	Controls (*n* = 11)
Age	41 (36–51)	36 (31–39)
Gender (Male: Female)	12∶5	6∶5
Ethnicity (Cauc. : Afr. Amer. : Hisp.)	4∶13∶0	7∶2∶2
CD4^+^ T cells/µl of blood	402 (294–530)	n.a.
Percent of pDC in total PBMC	0.25±0.04	0.57±0.07
Log_10_ HIV-1 RNA copies/ml blood	3.7 (1.7–4.7)	N/A
ART status (On: Off)	6∶11	N/A

All data are Median (Interquartile Range) except for % pDC in total PBMC, which is Mean ± Standard Error of Means; n.a.  = not available; N/A = not applicable; PBMC = peripheral blood mononuclear cells; ART = Antiretroviral therapy.

**Figure 2:**
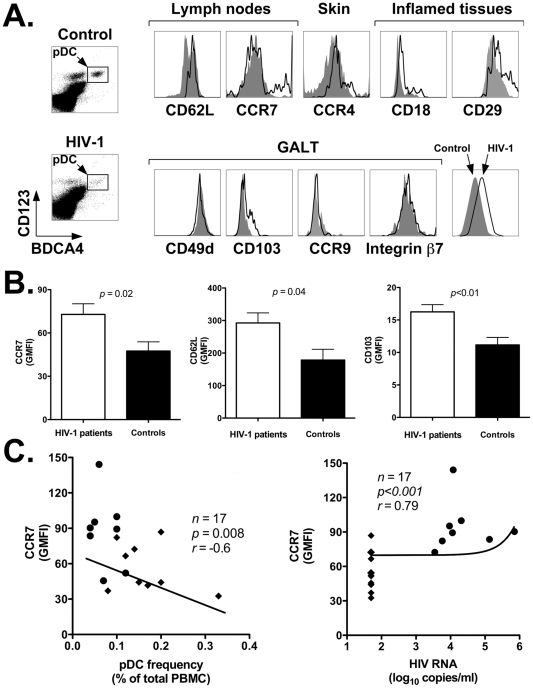
Increased expression of lymphoid tissue homing markers on circulating pDC of HIV-1 positive versus negative individuals. Panel A: representative flow cytometry plots showing expression of nine homing markers grouped by tissue specificity: CD62L and CCR7 (lymph nodes); CCR4 (skin); CD18 and CD29 (inflamed tissues); and CD49d, CD103, CCR9 and integrin β7 (GALT). Histogram plots were obtained by gating onto the live cell population as assessed by forward and side light scatter profile (not shown), and then onto the pDC population as identified by staining with anti-BDCA4 and anti-CD123 antibodies (rectangles within density plots). Top density plot and gray area histogram: healthy control; bottom density plot and black line histogram: HIV-1 patient. Panel B: summary results of CCR7, CD62L and CD103 expression on pDC of HIV-1 positive (*n* = 17) and negative individuals (*n* = 11). We assessed the Geometric Mean Fluorescence Intensity (GMFI) for each marker by flow cytometry (Materials and Methods). The panels show the Mean and SEM of the GMFI values determined for all individuals in the two study groups. Black bars: healthy controls; white bar: HIV-1 patients. Panel C: correlation analyses between CCR7 expression and frequency of pDC in PBMC of HIV-1 patients (left) and HIV-1 viremia (right). The line summarizing the data uses a linear model fitted on the logarithmic scale that expresses HIV-1 viremia. Closed circles (•): viremic patients; closed diamonds (⧫): aviremic patients.

To assess whether accumulation of pDC in LN of HIV-1 infected patients involved modulation of tissue homing markers, we analyzed the expression of several cell surface receptors on circulating pDC of HIV-1 positive and negative individuals. The markers we analyzed included: CD62L and CCR7 (lymph nodes); CCR4 (skin); CD18, CD29 (inflamed tissues); CD49d, CD103, CCR9, and integrin β7 (*lamina propria* and intraepithelial sites of the gut associated lymphoid tissue or GALT). Figure 2A shows representative flow cytometry plots obtained by staining PBMC from a representative healthy control (top density plot and gray area histogram plots) and an HIV-1 patient (bottom density plot and black line histogram plots) with antibodies directed to BDCA4 and CD123 (to identify pDC) in conjunction with antibodies against homing markers of interest. Table 3 summarized the results of our analyses for all HIV-1 patients and control individuals, and reports the *p* value determined with the nonparametric Wilcoxon rank-sum test. The data are expressed as the Mean (± SEM) of the Geometric Mean Fluorescence Intensity (GMFI) values for each cell surface marker determined by flow cytometry with cells from all individuals in the two study groups. As shown in the table, among all the markers analyzed, pDC of HIV-1 patients displayed a statistically significant upregulation of only three markers: CCR7, CD62L, and CD103 (CCR7: *p*<0.05, CD62L: *p*<0.05, CD103: *p*<0.01; see Table 3 and Figures 2B). The mean CCR7, CD62L and CD103 GMFI ± SEM in HIV-1 positive vs. negative individuals were: 101.2±29.3 vs. 47.5±6.3; 292.4±31.1 vs. 178.4±33.2; and 20.7±4.6 vs. 11.2±1.2, respectively. All other homing markers (CCR4, CD49d, integrin β7, CCR9, CD18 and CD29) did not show any statistically significant difference between the two study groups (*p*>0.05; see Table 3 and Figure 2A).

**Table 3:** Expression of homing markers on peripheral blood pDC.

Target Tissue	Homing marker	HIV-1 patients (*n* = 17)	Controls (*n* = 11)	*p* values
Lymph nodes	CCR7	101.2±29.3	47.5±6.3	<0.05
	CD62L	292.4±31.1	178.4±33.2	<0.05
Gut associated lymphoid tissue	CD49d	695.1±104.6	894.9±154.2	>0.05
	CD103	20.7±4.6	11.2±1.2	<0.01
	CCR9	14.4±1.3	15.3±0.9	>0.05
	Integrin β7	245.3±40.3	213.9±18.3	>0.05
Inflamed tissues	CD18	54.4±16.4	40.3±13.5	>0.05
	CD29	676.7±87.9	559.6±218.5	>0.05
Skin	CCR4	138.5±22.4	88.1±20.5	>0.05

All data are Mean ± Standard Error of Means of the Geometric Mean Fluorescence Intensity (GMFI) values determined for all individuals in each study group.

Interestingly, Figure 2C shows that CCR7 expression was higher on pDC of viremic than aviremic patients: it correlated inversely with their frequency in peripheral blood (*p* = 0.008, *r* = -0.6), and correlated directly with HIV-1 viremia (*p*<0.001, *r* = 0.79). Importantly, the line summarizing the data in Figure 2C uses a linear model fitted on the logarithmic scale that expresses HIV-1 viremia. Altogether, these results suggest that ongoing HIV-1 replication drives upregulation of CCR7 on pDC of HIV-1 patients. Thus, higher expression of CCR7 and CD62L might promote their relocation from peripheral blood to lymph nodes, which are the site of chronic viral replication.

### Increased migration of pDC from HIV-1 patients in response to CCR7 ligands

To determine whether higher CCR7 expression on pDC from peripheral blood of HIV-1 patients could mediate their migration from the blood to lymphoid organs, we used a transwell migration assay to determine the ability of pDC from a subset of HIV-1 positive (*n* = 11) and negative (*n* = 6) individuals to migrate in response to the CCR7 ligands, CCL19 and 21. Since CCR7 expression was found to correlate with HIV-1 viremia, the patients were divided into two groups: those with detectable (>50 copies/ml, off ART; *n* = 6) and those with undetectable HIV-1 RNA (<50 copies/ml, on ART; *n* = 5).

**Figure 3:**
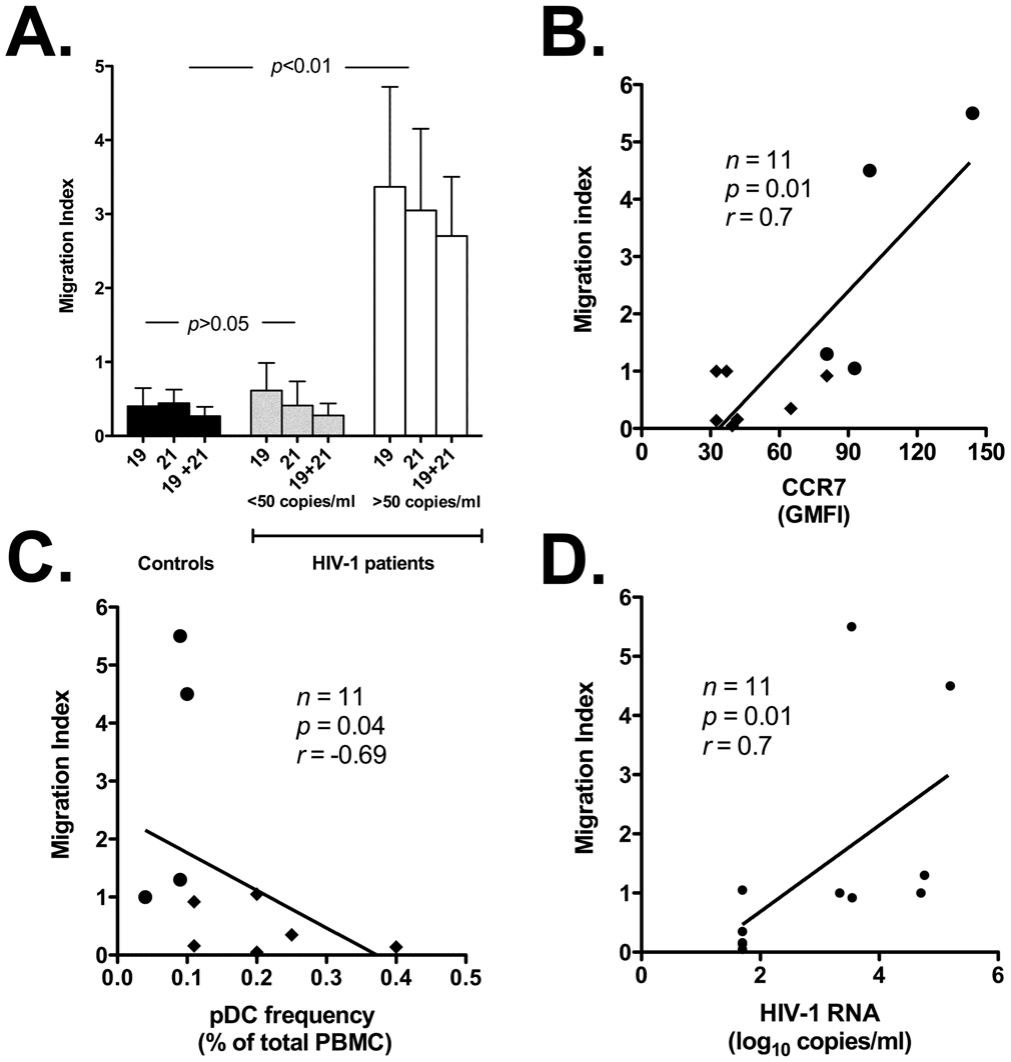
Higher migratory potential of circulating pDC from HIV-1 positive versus negative individuals in response to CCR7 ligands (CCL19 and CCL21). Panel A: migration index of circulating pDC in response to CCR7 ligands. Total PBMC were seeded in the upper chamber of a transwell plate, while medium containing CCL19 and CCL21 (alone and in combination) was placed in the lower chamber. Flow cytometry was used to score pDC found in the lower chamber in response to CCR7 ligands vs. medium alone. The figure shows Migration Indices (determined as described in Materials and Methods) for control individuals (black bars; *n* = 6) vs. aviremic HIV-1 patients (<50 copies/ml of HIV-1 RNA; gray bars; *n* = 5) vs. viremic patients (>50 copies/ml of HIV-1 RNA; white bars; *n* = 6). 19: treatment with CCL19; 21: treatment with CCL21; 19+21: treatment with CCL19 and CCL21. Panel B: direct correlation between Migration Index and CCR7 expression on pDC. Panel C: inverse correlation between Migration Index and pDC frequency in PBMC of HIV-1 patients. Panel D: direct correlation between Migration Index and HIV-1 viremia. Closed circles (•): viremic patients; closed diamonds (⧫): aviremic patients.

We observed that the net migration of pDC to CCL19 and 21 in HIV-1 positive individuals with detectable viral load was significantly greater than that of pDC from HIV-1 negative and positive individuals with undetectable viral load (*p*<0.01, Figure 3A). The migration index correlated directly with CCR7 expression on pDC (*p* = 0.01, *r* = 0.7; Figure 3B). Moreover, we found that the migration index of pDC correlated inversely with their frequency in PBMC (*p* = 0.04, *r* = −0.69; Figure 3C), and directly with HIV-1 viremia (*p* = 0.01, *r* = 0.7; Figure 3D).

We conclude that CCR7 upregulation on pDC of HIV-1 patients translates into an increased migratory potential in *ex vivo* assays. These results are in agreement with the ones described above, and further support the possibility that decreased pDC numbers in peripheral blood are due to relocation to lymph nodes.

### Abnormal cell surface marker expression profile and increased IFNα expression in lymph node-homed pDC of HIV-1 patients

Previous studies showed that pDC isolated from peripheral blood of HIV-1 patients produced lower levels of IFNα upon *ex vivo* stimulation with reference viruses, which was initially attributed to a functional defect of these cells due HIV-1 infection [17], [46]. However, subsequent studies demonstrated that *in vitro* stimulation does not faithfully reflect the ability of pDC to produce IFNα *in vivo* [47]. Indeed, we have demonstrated that circulating pDC of HIV-1 patients express markedly higher levels of IFNα mRNA and protein [28]. To address the possibility that chronic HIV-1 replication in lymph nodes may affect the activation, maturation and function of pDC, we analyzed these cells by flow cytometry for the expression of the activation and maturation markers CD40, BCDA2, CD83 and CD86, as well as the steady state IFNα secretion [38], [48], [49], [50].

**Figure 4:**
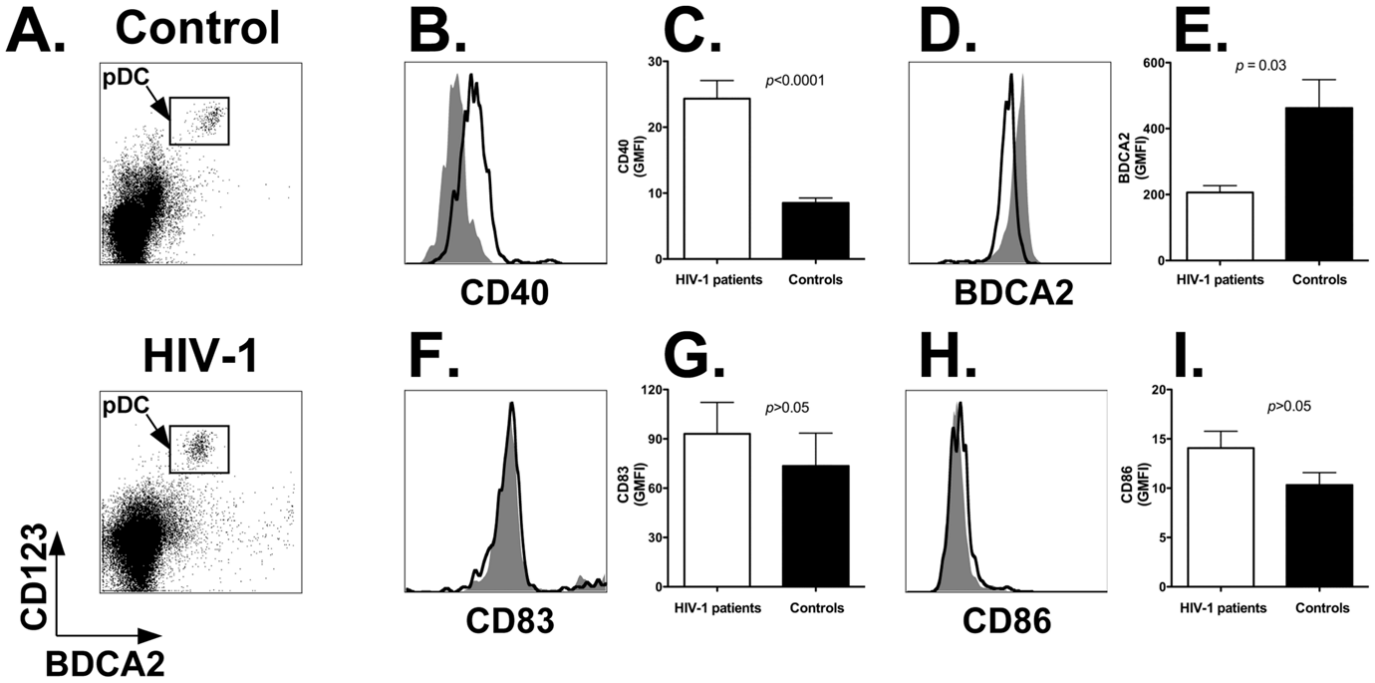
Activated but immature phenotype of lymph node-homed pDC from HIV-1 positive versus negative individuals. Panel A: identification of pDC populations in LNMC of a representative control (top panel) and HIV-1 positive (bottom panel) individual. LNMC were stained with anti-BDCA2 and anti-CD123 antibodies; analyses were carried out by gating on the live cell population as determined by forward and side scatter light profiles. Panels B, D, F and H: flow cytometry plots showing expression levels of activation (CD40 and BDCA2) and maturation (CD83 and CD86) markers on lymph node-homed pDC from a representative HIV-1 patient and control individual. Histogram plots were obtained by gating onto the live cell population as assessed by forward and side light scatter profile (not shown), and then onto the pDC population as identified by staining with anti-BDCA2 and anti-CD123 antibodies (as shown in density plots). Gray area: healthy control; black line: HIV-1 patient. Panels C, E, G, and I: expression levels of CD40, BDCA2, CD83 and CD86 on lymph node-homed pDC of control individuals (black bars; *n* = 11) and HIV-1 patients (white bars; *n* = 18). We assessed the Geometric Mean Fluorescence Intensity (GMFI) values for each marker (Materials and Methods). Panels show the Mean and SEM of the GMFI values determined with cells from all individuals in the two study groups.

Whole LNMC preparations were stained with antibodies directed to BDCA2 and CD123 (to identify the pDC population; Figure 4A; top panel: healthy control; bottom panel: HIV-1 patient) in combination with antibodies directed to CD40, CD83 or CD86. Figure 4 shows representative flow cytometry histogram plots obtained by staining LNMC from a healthy control (gray area) and an HIV-1 patient (black line) with anti-CD40 (panel B), -BDCA2 (panel D), -CD83 (panel F), and -CD86 (panel H) antibodies, respectively. Summary results shown in Figure 4C demonstrate that CD40 expression was significantly upregulated on pDC of HIV-1 patients (white bar) compared to healthy subjects (black bar; *p*<0.0001). The mean GMFI ± SEM was 26.2±3.5 for HIV-1 patients vs. 7.8±1.3 for uninfected persons (Table 4). In addition, Figure 4E shows that BDCA2 expression was significantly lower on pDC of HIV-1 patients (*p*<0.03): the mean GMFI ± SEM was 206.2±20.8 vs. 462.5±85.9 for controls (Table 4). However, we found no difference (*p*>0.05) in the expression levels of CD83 and CD86 between HIV-1 patients and healthy controls: 99.7±26.5 vs. 77.8±34.6; and 14.7±2.4 vs. 9.9±2.3 (Figure 4G and 4I; Table 4). The increase in CD40 expression did not correlate with proviral HIV-1 DNA content (*r* = 0.4, *p*>0.05), but correlated directly with HIV-1 viremia (*r* = 0.62, *p* = 0.02; (data not shown).

**Table 4:** Features of lymph node-homed pDC.

Feature	Marker	HIV-1 positive (*n* = 18)	Controls (*n* = 11)	*p* values
Activation	CD40	26.2±3.5	7.8±1.3	<0.0001
	BDCA2	206.2±20.8	462.5±85.9	<0.03
Maturation	CD83	99.7±26.5	77.8±34.6	>0.05
	CD86	14.7±2.4	9.9±2.3	>0.05
Function	IFNα	66.7±11.3	40.0±8.9	<0.01
Death	Annexin V	15.5±1.5	8.9±1.9	= 0.01

Data are Mean ± Standard Error of Means of the Geometric Mean Fluorescence Intensity (GMFI) values determined for all individuals in each study group. For Annexin V, data are Mean ± Standard Error of the percent Annexin V positive values determined for all individuals in each study group.

**Figure 5:**
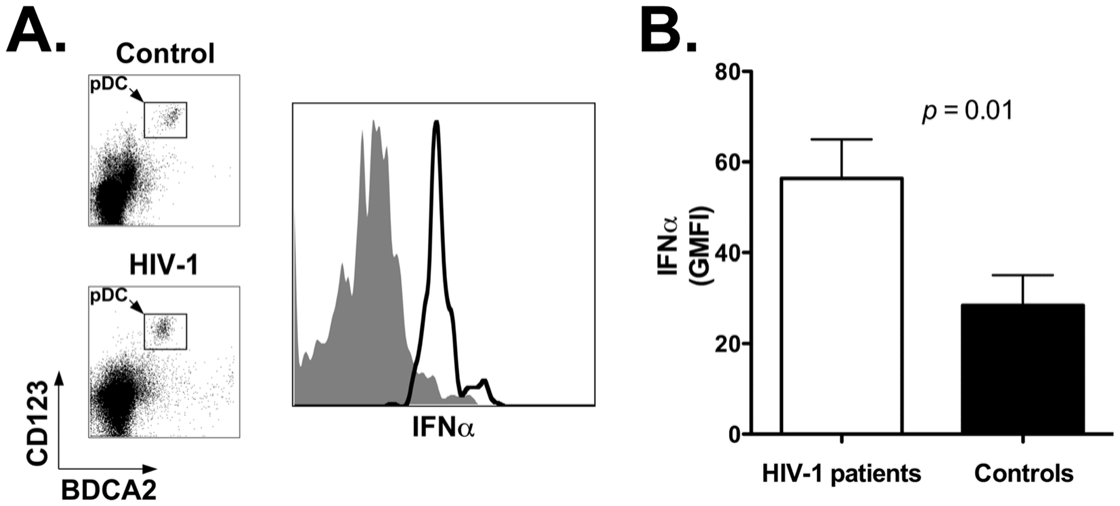
Increased IFNα expression by pDC in lymph nodes of HIV-1 patients. Panel A: flow cytometry plot showing expression of IFNα by lymph node-homed pDC from a representative healthy control and an HIV-1 patient. Histogram plot was obtained by gating onto the live cell population as assessed by forward and side light scatter profile (not shown), and then onto the pDC population as identified by staining with anti-BDCA2 and anti-CD123 antibodies. Top density plot and gray area histogram: healthy control; bottom density plot and black line histogram: HIV-1 patient. Panel B: summary results for expression levels of IFNα by lymph node-homed pDC of healthy controls (black bar; *n* = 11) and HIV-1 patients (white bar; *n* = 18). We assessed the Geometric Mean Fluorescence Intensity (GMFI) value with cells from all individuals in the two study groups (Materials and Methods). Panel shows the Mean and SEM of the GMFI values.

Next we analyzed baseline IFNα secretion by pDC using a flow cytometry-based assay that detects the cytokine following secretion by the producer cell. Pilot experiments showed that IFNα secretion under our assay conditions is unaffected by the process of cell freezing and thawing (not shown). Figure 5A shows representative results obtained with cells from a healthy control (top density plot and gray area histogram plot) and an HIV-1 patient (bottom density plot and black line histogram plot). The summary results for all individuals in each study group show that unstimulated pDC of HIV-1 patients (white bar) secrete significantly higher amounts of IFNα compared to control individuals (black bar; *p* = 0.01): the mean GMFI ± SEM for the two study groups was 66.7±11.3 vs. 40.0±8.9, respectively (Figure 5B and Table 4). These results demonstrate that lymph node-homed pDC of HIV-1 patients present activated but immature phenotype, and secrete elevated amounts of IFNα.

### Lymph node-homed pDC of HIV-1 patients undergo apoptosis at higher rate

As shown above, our results indicate that the decline of circulating pDC in HIV-1 patients is associated with increased expression of functional lymph node homing markers. We also found increased frequencies of pDC in lymph nodes of HIV-1 patients, particularly those with higher viral loads. Next, we sought to determine whether there might be underlying mechanisms that compensate the migration of pDC to lymph nodes in HIV-1 patients, thereby offsetting in part accumulation of these cells.

**Figure 6:**
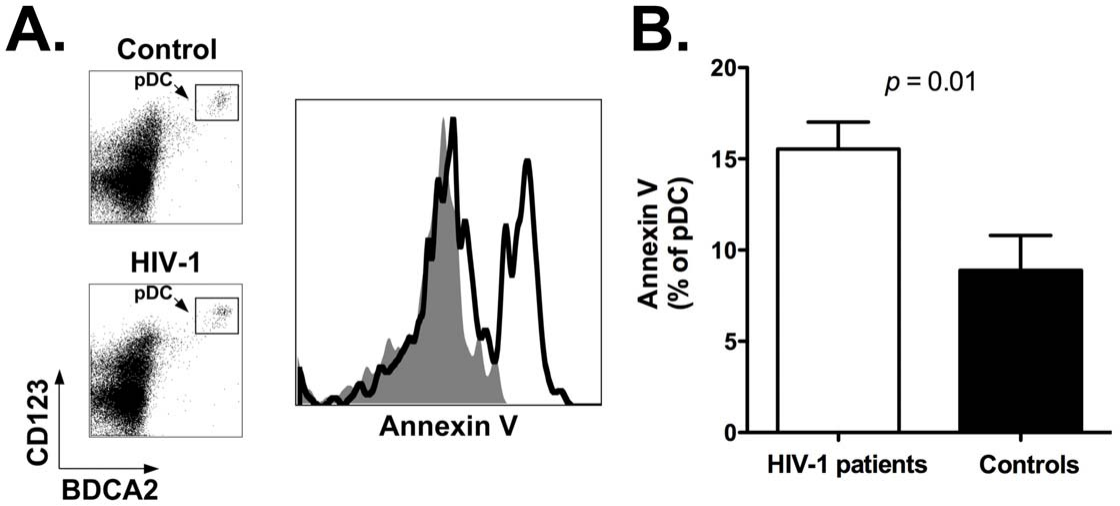
Higher rates of cell death by pDC in lymph nodes of HIV-1 patients. Panel A: flow cytometry plot showing Annexin V staining of lymph node-homed pDC from a representative HIV-1 patient and control individual. Histogram plots were obtained by gating onto the live cell population as assessed by forward and side light scatter profile (not shown), and then onto the pDC population as identified by staining with anti-BDCA2 and anti-CD123 antibodies. Top density plot and gray area histogram: healthy control; bottom density plot and black line histogram: HIV-1 patient. Panel B: summary results for expression levels of Annexin V by lymph node-homed pDC of healthy controls (black bar; *n* = 11) and HIV-1 patients (white bar; *n* = 18). We determined the percentage of Annexin V positive pDC from all individuals in the two study groups (Materials and Methods). Panel shows the Mean and SEM values of the percent Annexin V positive pDC.

A potential mechanism involved in this phenomenon could be a higher rate of pDC death after relocation to lymph nodes [33]. Therefore, uncultured, non-stimulated LNMC from HIV-1 patients and controls were stained with antibodies directed to BDCA2 and CD123 (to identify the pDC population) in combination with Annexin V, which allows the identification of cells undergoing apoptosis. As shown in Figure 6B, the mean percentage of Annexin V positive pDC (± SEM) was significantly higher for HIV-1 positive (white bar) than negative individuals (black bar; *p* = 0.01): 15.5±1.5 vs. 8.9±1.9 (Table 4). Figure 6A shows plots representative of a healthy control (top density plot and gray area histogram plot) and an HIV-1 patient (bottom density plot and black line histogram plot). Since cells from HIV-1 positive individuals are more susceptible to apoptosis and may be lost at a higher rate during the freezing-thawing process compared to cells of healthy individuals, it is conceivable that the higher frequency of Annexin V-positive pDC in HIV-1 patients may be somewhat underestimated.

### Correlation analyses

**Figure 7:**
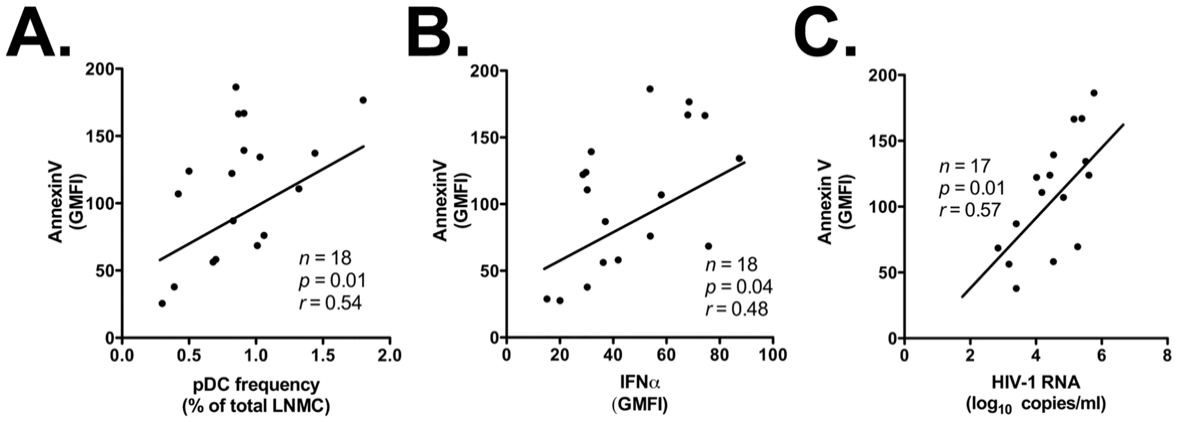
Increased rates of pDC apoptosis correlate with pDC frequency, IFNα expression by LN-homed pDC, and HIV-1 viremia. Panels A, B and C: direct correlation between intensity of Annexin V staining on LN-homed pDC of HIV-1 patients and their frequency in LNMC, their ability to express IFNα, and the HIV-1 viral load in peripheral blood, respectively.

Based upon our nonparametric correlation analyses, IFNα secretion was not associated with pDC frequency in LNMC, CD40 expression on lymph node-homed pDC, HIV-1 proviral DNA in lymph nodes, or HIV-1 blood viremia (*p*>0.05; data not shown). A possible explanation for these results – which is further addressed in the Discussion – is that pDC express IFNα in response to HIV-1 virions and/or proteins trapped in the intercellular space in lymph nodes. In addition, while we did not find any significant correlation between Annexin V staining and HIV-1 proviral DNA (data not shown), Annexin V staining correlated significantly with the frequency of pDC in lymph nodes (*r* = 0.54, *p* = 0.01; Figure 7A), IFNα secretion by pDC in lymph nodes (*r* = 0.48, *p* = 0.04; Figure 7B), and HIV-1 blood viremia (*r* = 0.57, *p* = 0.01; Figure 7C).

Altogether, the results presented in this report demonstrate that during HIV-1 infection pDC relocate to lymph nodes and secrete higher amounts of IFNα than their counterparts in uninfected individuals. Concurrently, pDC undergo cell death at significantly higher rate, which may in part offset their accumulation in lymph nodes.

## Discussion

In the present report we investigated fate, activation, maturation, and function of plasmacytoid dendritic cells (pDC) during HIV-1 disease. Since most of the HIV-1 positive blood donors and all LN donors enrolled in our study were treatment-naïve at time of sample collection, the effects of anti-retroviral therapy on viral replication, immune cell distribution, activation and function are not expected to influence significantly our results.

The fate of pDC in the course of HIV-1 infection has been a matter of intense debate. Soon after the identification of CD4^+^Lin^–^CD11c^–^BDCA2/4^+^CD123^+^ plasmacytoid dendritic cells as the major IFNα-producing cell type in humans upon virus infection [15], [50], several groups reported the gradual decline of pDC in peripheral blood of HIV-1 patients [16], [18], [19]. These results were attributed to depletion following direct virus infection [19], [36], [37]. However, subsequent studies suggested that the decline of pDC in peripheral blood does not reflect a systemic depletion of these cells [28], [38], [39], and may involve relocation to lymphoid tissues, but failed to provide a mechanism that could explain the observations [38]. The present report shows for the first time that pDC accumulate in lymph nodes of HIV-1 patients, particularly those with highest viral loads. A recent study by Panda *et al*. showed that circulating pDC decline substantially in older compared to younger individuals [51]. In our study, there was no statistically significant difference in the median age of uninfected and infected blood donors. Therefore, age was not a factor in the decline in circulating pDC in HIV-1 infected individuals compared to healthy controls. As indicated in the Materials and Methods, the studies described in the present report were carried out with cryopreserved cell preparations. In order for the freezing-thawing process to account for the depletion or accumulation of pDC in peripheral blood or lymph nodes, respectively, of HIV-1 patients vs. control individuals that we describe here, one would have to postulate that: a) to explain a selective change in pDC frequency compared to other cells populations (T cells, B cells, NK cells, myeloid cells, etc), pDC would have to be affected by freezing-thawing differently than other cell types present in the preparations being analyzed; and b) to explain a selective depletion of pDC in peripheral blood and a selective accumulation in lymph nodes, pDC would have to be affected by freezing-thawing in opposite fashion in PBMC vs. LNMC preparations. While it is conceivable that freezing-thawing may kill cells from viremic HIV-1 patients more than cells from aviremic patients or healthy individuals, we believe the postulates outlined above to represent an unlikely scenario. Thus, we do not believe that the freezing-thawing process influenced, skewed or altered our studies in a way to lead to “artifactual” results.

Concurrently, we found that pDC in peripheral blood of HIV-1 patients express significantly higher levels of the lymph node homing markers, CCR7 and CD62L. Indeed, we showed that pDC of viremic HIV-1 patients responded more potently to CCR7 ligands – CCL19 and CCL21 – in *ex vivo* migration assays under conditions that minimize cell differentiation. Altogether, these results provide a mechanism for the accumulation of pDC in LN. We found a strong correlation between HIV-1 viremia, CCR7 expression, lower frequency in PBMC, and migratory potential in *ex vivo* assays. Indeed, exposure of pDC to infectious or noninfectious HIV-1 particles *in vitro* leads to upregulation of functional CCR7 [52]. Moreover, previous studies by Desai *et al*. and by Dillon *et al*. failed to observe an up-regulation of CCR7 on circulating pDC of HIV-1 patients [38], [53]. The inconsistency between our findings and those of those two studies may be due to a number of factors. For instance, Desai *et al.* focused their analysis on pediatric patients under therapy, while our study involved adult patients most of which were not undergoing ART. On the other hand, Dillon *et al*. used mean fluorescence intensity (MFI) to analyze CCR7 expression, while our study employed geometric mean fluorescence intensity (GMFI). Since CCR7 expression does not follow a normal distribution, and since its expression has a dynamic range of 2-3 logs, GMFI gives a more accurate estimate of the central tendency in the data sets than MFI. This conclusion is also supported by our functional studies, which showed that pDC of viremic HIV-1 patients display increased migratory potential in *in vitro* assays. Therefore, our studies are the first to demonstrate that the decline of pDC in peripheral blood of asymptomatic HIV-1 patients does not simply reflect a systemic cell loss, but rather can be explained – at least in part – with redistribution to peripheral lymph nodes in response to viral replication. Our results reflect closely the situation observed in pathogenic SIV infection of non-natural hosts. Several studies showed a sharp decline of circulating pDC during the acute phase of SIV infection in macaques, which persists throughout the infection [33], [34], [54], [55]. Concurrently, pDC were found at higher frequency in LN of infected animals [54], [55], only to decline with the onset of AIDS through a mechanism involving apoptosis [33]. The acute phase of non-pathogenic SIV infection in natural hosts (e.g. sooty mangabeys and African green monkeys) also involves a decline of circulating pDC, which initially relocate to lymph nodes, and then return to the circulation following the onset of the chronic phase [35]. Therefore, pDC dynamics sharply discriminate pathogenic and non-pathogenic lentiviral infection in humans and animal models. Our studies also found that circulating pDC of HIV-1 patients express higher levels of CD103 (also known as integrin αE), which – in complex with integrin β7 – mediates cell redistribution to the intraepithelial sites and *lamina propria* of the GALT. Evidence of higher levels of CD103 on pDC of HIV-1 patients may also indicate relocation of these cells to lymphoid tissues lining the gut mucosa. This hypothesis will be addressed in future studies.

In this report we also assessed the activation, maturation and function of pDC in lymph nodes of HIV-1 patients compared to healthy controls. Our results demonstrate that pDC in LN of HIV-1 donors display an altered cell surface expression profile of activation/maturation markers with higher CD40, lower BDCA2, and stable CD83 and CD86 levels. A previous report found that interdigitating dendritic cells in lymph nodes of HIV-1 patients also express higher CD40 and lower CD86 levels [56]. In addition, that study found a higher percentage of IFNα-expressing cells in lymph nodes of HIV-1 positive individuals. The decline in BDCA2 expression on pDC of HIV-1 patients is relevant for multiple reasons [57]. First, BDCA2 is a class II C-type lectin involved in antigen capture and presentation [50], [58]. Second, pDC stimulated through BDCA2 promote CD4^+^ T cell proliferation and differentiation toward Th1 effector cells [59]. Third, BDCA2 stimulation leads to decreased IFNα expression by pDC [58]. Thus, lower BDCA2 levels on pDC of HIV-1 patients are consistent with the notion that these cells have reduced capacity to function as antigen presenting cells and to stimulate CD4^+^ T cell proliferation, while retaining the ability to express IFNα. Indeed, we demonstrated that pDC from LN of HIV-1 patients secrete higher amounts of IFNα by using a flow cytometry-based assay in the absence of exogenous stimuli that may yield artifactual results [47]. Also, our previous studies showed that IFNα expression by circulating pDC was increased in HIV-1 patients both at the RNA and protein level, but did not correlate with HIV-1 viral load in blood [28], [30]. A recent study also showed that pDC of women express more IFNα than pDC of men [60]. Since in our study the cohort of HIV-1 positive lymph node donors included only men, it is possible that a cohort including both men and women would have evidenced an even more dramatic increase in IFNα expression by lymph node-homed pDC during HIV-1 infection. In the present report, we did not observe any correlation between IFNα expression by LN-homed pDC and HIV-1 viremia. Thus, active virus replication appears to lead to higher CCR7 expression and pDC migration to lymph nodes, but not to higher IFNα expression by lymph node-homed pDC. However, several studies showed that pDC can be triggered to express IFNα following exposure to both infectious and non-infectious HIV-1 as well as by HIV-1 proteins [61], [62], suggesting that viral particles and/or proteins trapped in the intercellular space of lymph nodes may be involved in promoting IFNα expression by pDC. Indeed, recent studies found that viral gag proteins persist on the surface of the FDC network over prolonged periods of time even during controlled viral replication in the presence of HAART [63]. Alternatively, this may be the consequence of events set in motion during HIV-1 infection, but not sustained by ongoing viral replication. Once again, parallel studies conducted in pathogenic versus non-pathogenic SIV models underscore the relevance of our findings in the context of HIV-1 infection. Indeed, pDC express copious amounts of IFNα during the acute and chronic phase of SIV infection in macaques, both in peripheral blood and in lymphoid tissues [54], [64]. By converse, pDC express IFNα in lymph nodes during the acute phase of SIV infection in the natural host, but IFNα levels returns to baseline values during the chronic phase of the non-pathogenic infection [35]. Moreover, the amplitude of the IFNα response to SIV infection in natural hosts during the acute phase is reduced compared to non-natural hosts [29], [35], reflecting a lower responsiveness of pDC from natural hosts to TLR7 and TLR9 stimuli [29].

Persistent activation of the immune system in the face of impaired immune responses is a hallmark of pathogenic lentiviral infection, and is a potent predictor of disease progression [2], [3], [4], [5], [6]. By contrast, non-pathogenic SIV infection is characterized by normal levels of immune activation despite vigorous viral replication [65]. The mechanism(s) at play in this phenomenon have been investigated, but remain elusive. Recently, several studies that bacterial translocation from the lumen of the gastro-intestinal tract following HIV-1 and SIV-mediated depletion of CD4^+^ T cells in the GALT might be involved [66]. However, this hypothesis has been questioned because of the lack of bacterial translocation in non-pathogenic SIV infection despite CD4^+^ T cell depletion in the GALT [66], [67]. Recently, two reports suggested that over-expression of IFNα may be involved in driving persistent immune activation during HIV-1 infection. Sedaghat *et al.* reported that activated CD4^+^ T cells from peripheral blood of untreated HIV-1 patients are in a hyper-proliferative state under the modulation of IFNα [31]. In addition, studies by Mandl *et al.* suggested that production of IFNα by pDC in SIV-infected non-natural hosts may drive the chronic immune activation and dysfunction leading to AIDS [29]. However, neither study addressed the mechanism underlying this phenomenon.

In summary, we showed that untreated HIV-1 infection is characterized by elevated expression of CCR7 and CD62L on plasmacytoid dendritic cells, which relocate to lymph nodes, acquire an activated but immature phenotype, and express elevated amounts of IFNα before undergoing cell death. Our results are in line with other reports suggesting a potential role for IFNα in promoting chronic immune activation during HIV-1 and SIV infection. Further studies into this phenomenon may shed new light on the mechanisms of HIV-1 pathogenesis.
